# Temperature Influences Antimicrobial Resistance and Virulence of *Vibrio parahaemolyticus* Clinical Isolates from Quebec, Canada

**DOI:** 10.3390/pathogens14060521

**Published:** 2025-05-23

**Authors:** Feriel C. Mahieddine, Annabelle Mathieu-Denoncourt, Marylise Duperthuy

**Affiliations:** 1 Département de Microbiologie, Infectiologie et Immunologie, Faculté de Médecine, Université de Montréal, Montréal, QC H3T 1J4, Canada; 2 Centre d’Innovation Biomédicale, Faculté de Médecine, Université de Montréal, Montréal, QC H3T 1J4, Canada

**Keywords:** *Vibrio parahaemolyticus*, virulence, biofilm, motility, hemolysin, antimicrobial resistance, temperature

## Abstract

Climate change and ocean warming have a huge impact on microbial communities, leading to an increased prevalence of *Vibrio parahaemolyticus* infections in northern America. *V. parahaemolyticus* is an inhabitant of aquatic environments and is associated with fishes and shellfishes, particularly with oysters. It causes gastrointestinal infection through consumption of contaminated seafood, as well as wound infections or septicemia. Temperature is known to affect virulence and persistence factors in *V. parahaemolyticus*. In this study, twenty clinical strains isolated form sick patients in Quebec, Canada, were characterized for persistence and virulence factor production at different temperatures to assess the impact of a switch from sea water to the human body on them. Their capacity to produce biofilm, hemolysins, and membrane vesicles as well as their motility and antibiotic resistance at 20 °C and 37 °C were assessed. Our results revealed that while temperature had little effect on vesicle production, it significantly influenced their growth, antimicrobial resistance, biofilm formation, and motility. Additionally, all *V. parahaemolyticus* strains produced hemolysins at 37 °C but not under environmental conditions.

## 1. Introduction

*Vibrio* are Gram-negative bacteria that reside in relatively warm and low-salinity marine environments [1]. They are commonly associated with a wide range of aquatic animals, including fish, crustaceans, and mollusks. Among the *Vibrio* genus, *V. cholerae*, *V. parahaemolyticus*, *V. vulnificus*, and *V. alginolyticus* are the primary species with strains that cause disease in humans [2]. *Vibrio* are the only foodborne pathogens with an increasing incidence in the United States, which makes them an important concern [1]. In this country, *Vibrio* comprise the most reported cause of seafood-associated outbreaks, with *V. parahaemolyticus* being the species most commonly isolated from affected patients [3,4]. Cases of *V. parahaemolyticus* infection have increased annually over the last decade [4,5,6,7]. It is indeed estimated to be responsible for more than 92,000 infections [8], and it represents 40% of all the reported *Vibrio* infections, called vibriosis, each year [3,4]. *V. parahaemolyticus* is also responsible for up to 19% of all the non-foodborne *Vibrio* infections [9]. Vibriosis have been under surveillance by the Centers for Disease Control and Prevention since 2007, when the number of cases first became noticeable [5].

While the United States (CDC) and British Columbia (BC Center for Disease Control) have surveillance programs for *Vibrio* and *V. parahaemolyticus* infections, such a program does not exist for the province of Quebec or for Canada at a national level. In Canada, important *V. parahaemolyticus* outbreaks occurred in 1997, 2015, and 2020, infecting more than 209, 82, and 23 people, respectively [10,11,12]. The two first outbreaks were linked to the consumption of shellfishes from British Columbia coastal waters, while the 2020 outbreak was linked to shellfishes from Atlantic Canadian coastal waters and touched seven people from the province of Quebec [10].

*V. parahaemolyticus* infections mostly occur by ingestion of contaminated undercooked or raw seafood, like oysters. Besides the acute gastroenteritis that *V. parahaemolyticus* can cause, it can be responsible for wound infections after exposure to contaminated water [2,13]. On rare occasions, *V. parahaemolyticus* can cause a sepsis in patients with underlying health conditions [14]. Amongst *V. parahaemolyticus*’s many virulence factors [15], the thermostable direct hemolysin (TDH) and TDH-related hemolysin (TRH) are the most important, inducing inflammatory gastroenteritis [16,17,18]. Their production is associated with pathogenic strains [19], although environmental strains can also encode for these toxins [20]. Its virulence factors also include two type III secretion systems [21], adhesion [22,23] and motility [24]. *V. parahaemolyticus* possesses two flagellar systems consisting of a single polar flagellum and lateral flagella. They are responsible for motility, adhesion to surfaces, and biofilm formation and for persistence in oysters [22,25]. *V. parahaemolyticus* can form biofilm—structured microbial communities embedded in a matrix—on seafood, enhancing its persistence and posing a threat to food safety [22,25].

Generally, *V. parahaemolyticus* outbreaks are seasonal and are associated with warmer water temperature, promoting bacterial spread [2,26]. For instance, the 2015 outbreak was associated with a historically high water temperature in the area [26,27]. Climate change is one of the main issues of the 21st century, affecting not only human populations but also ecosystems and their microbial inhabitants [28,29]. One of the consequences of climate change is the rise in ocean temperature, promoting the emergence of pathogenic bacteria and the expansion of their habitat area due to reduced biodiversity and altered environmental conditions [28,30]. The effect of climate change on marine bacteria extends beyond higher temperature, encompassing lower salinity, increased acidity, altered water circulation patterns, and rising sea water levels [28,30]. As marine bacteria, *Vibrio* are exposed to ocean warming, which represents an increasing concern regarding risks of infection by pathogenic marine bacteria [1,31,32,33]. Ocean warming has been linked to the increase in *Vibrio* infections’ prevalence [30,31,32,33,34], and the portrait is likely to worsen, including in Canada, making it urgent to understand *V. parahaemolyticus*’s virulence and persistence.

Temperature is known to be a modulator of virulence in *V. parahaemolyticus*, especially during the shift from the environment to the human body [17,22]. A collection of 20 clinical strains of *V. parahaemolyticus* isolated from sick patients in Quebec from August 2018 to August 2022 was provided by the Laboratoire de santé publique du Québec (LSPQ, a part of Institut National de Santé Publique du Québec). In this study, they were characterized for resistance and persistence traits at different temperatures, representing water and human body temperatures, to assess how temperature affects their virulence and persistence. Their resistance to antimicrobials was determined, and it showed a better global resistance at 20 °C than at 37 °C. Vesiculation at 20 °C and 37 °C were comparable. *V. parahaemolyticus* was generally more motile at 37 °C, which correlates with a lower capacity to form biofilm than at 20 °C. The hemolysis capacity was compared, and it showed that all the strains produce hemolysins at 37 °C but not at 20 °C. The presence of the major hemolysins *tdh* and *trh* was assessed by PCR and revealed that 95% of the strains encode for at least one of them, while one strain contained neither of them. The temperature significantly modified the phenotypes exhibited by *V. parahaemolyticus*, and our results could help to understand this emerging pathogen.

## 2. Materials and Methods

### 2.1. Strains

A collection from LSPQ (a part of Institut National de Santé Publique du Québec) of 20 pathogenic strains of *V. parahaemolyticus* isolated from patients in Quebec from 2018 to 2022 was used for this study, and the strains are listed in Table 1. *V. parahaemolyticus* strains were grown in LB2%NaCl (10 mg mL^−1^ tryptone (Termo Fisher™, Waltham, MA, USA), 5 mg mL^−1^ yeast extract (Thermo Fisher™, Waltham, MA, USA), 20 mg mL^−1^ NaCl (Thermo Fisher™, Waltham, MA, USA) or on LB2%NaCl agar plates (12 mg/mL agar) at 37 °C.

### 2.2. Growth Curves and Minimal Inhibitory Concentrations

*V. parahaemolyticus* was grown at 37 °C to an optical density of 600 nm (OD_600nm_) of 0.3, then were further diluted by 1:30,000 in LB2%NaCl and distributed in 96-well plates with decreasing concentrations of antimicrobial peptides and antibiotics. Bacterial growth was measured by reading the OD_600nm_ after 18 h of incubation at 20 °C and 37 °C. The minimal inhibitory concentration was defined as the smallest concentration inhibiting 100% of the bacterial growth (OD_600nm_ = 0).

One hundred microliters of overnight cultures of *V. parahaemolyticus* diluted by 1:20 in fresh LB2%NaCl were distributed in a 96-well plate, which was further incubated at 20 °C and 37 °C with agitation. The growth curves were obtained by reading the OD_600nm_ every hour using a SpectraMax ID3 plate reader (Molecular Devices, San Jose, CA, USA). Bacterial suspensions were diluted in PBS and spread on LB2%NaCl agar plates for bacterial counts. Data were obtained from three independent experiments in technical triplicates.

### 2.3. Biofilm Formation Assays

Biofilm formation was assessed as described before [35]. Briefly, bacteria were grown for 16 h in LB2%NaCl at 37 °C then diluted by 1:100 in fresh medium. Bacteria were distributed in a 96-well plate and incubated at 20 °C and 37 °C for 48 h without shaking. The biofilms were washed with sterile water, dried, and stained with 0.1% crystal violet for 10 min. After washing, the biofilms were dissolved in 100 µL of 30% acetic acid. The optical density at 595 nm (OD_595nm_) of the suspension was measured using a plate reader (Molecular Devices, San Jose, CA, USA) to quantify the biofilm biomass. The values were normalized on the OD_600nm_ of the planktonic phase.

### 2.4. Hemolysin Production and Detection of tdh and trh by PCR

*V. parahaemolyticus* was grown in LB2%NaCl for 16 h at 37 °C. Three microliters of the bacterial suspension was spotted on sheep blood agar plates (Oxoid, Nepean, ON, Canada). The plates were incubated at 20 °C or 37 °C for 48 h. The diameter of the colony and of the hemolysis zone were measured. The colony diameter was subtracted from the diameter of the hemolysis zone. The experiment was repeated 4 times in independent experiments.

The presence of *tdh* and *trh* in the clinical strains was determined by PCR with commonly used sets of primers [20,36]. *tdh* was amplified with L-*tdh*: 5′-gtaaaggtctctgacttttggac-3′ and R-*tdh* 5′-tggaatagaaccttcatcttcacc-3′, and *trh* was amplified with L-*trh*: 5′-ttggcttcgatattttcagtatct-3′ and R-*trh*: 5′-cataacaaacatatgcccatttcc g-3′, using DNA Taq Polymerase from New England Biolabs (Whitby, ON, Canada). The cycle consisted of an initial denaturation step of 30 s at 95 °C followed by 30 cycles of amplification consisting of a denaturation step of 20 s at 95 °C, primer annealing at 47 or 45 °C for 20 s (for *tdh* and *trh*, respectively), and extension at 68 °C for 30 s. A final extension step of 5 min at 68 °C was added. The sample migrated on 1% agarose gel with RedSafe™ Nucleic Acid Staining Solution. The experiment was repeated three times to ensure the absence of false positives or negatives.

### 2.5. Swimming Motility Assays

Swimming motility assays were performed as previously described [37,38], with slight modifications. Overnight cultures of bacteria were spotted on LB2%NaCl plates containing 0.3 or 1.2% agar. They were incubated for 24 h at 20 °C or 37 °C. The diameter of the bacterial growth was measured at 24 h. The relative diameter was calculated by dividing the diameter on motility agar (0.3%) by the diameter on agar plates (1.2%) for a strain at the same temperature. The experiment was repeated in six independent experiments.

### 2.6. Membrane Vesicle Production

Membrane vesicle production in the culture supernatant was assessed using the fluorescent marker FM1-43 (*N*-(3-Triethylammoniumpropyl)-4-(4-(Dibutyl amino) Styryl) Pyridinium Dibromide, Invitrogen, Waltham, MA, USA). *V. parahaemolyticus* clinical strains were grown in 150 µL of LB 2% NaCl in 96-well plates at 20 and 37 °C with agitation for 16 h. MO10 and its hypervesiculating variants V2 and V8 were used as control [38]. The OD_600nm_ of the cultures was measured to assess bacterial growth, then the cultures were transferred into round-bottom plates and centrifuged at 1500× *g* for 30 min. Fifty microliters of the supernatant were transferred into a 96-well plate and FM1-43 was added at a final concentration of 10 µg/mL. The plates were incubated for 5 min in the dark, and the fluorescence at 479/579 nm was measured in SpectraMax ID3 plate reader (Molecular Devices, San Jose, CA, USA). The relative fluorescence was calculated by dividing the absolute fluorescence values by the bacterial growth values (OD_600nm_). The experiment was conducted in duplicates and repeated in 5 independent experiments.

### 2.7. Statistical Analysis

All data are expressed as mean ± SD and were analyzed for significance using GraphPad Prism (version.9.5.1). Student’s *t*-tests were used to compare conditions between 2 groups. One-way ANOVA was used for multiple group comparison. A result was considered as significant when the *p* value < 0.05 (*).

## 3. Results

### 3.1. V. parahaemolyticus Grows Faster at 37 °C than at 20 °C

**Figure 1:**
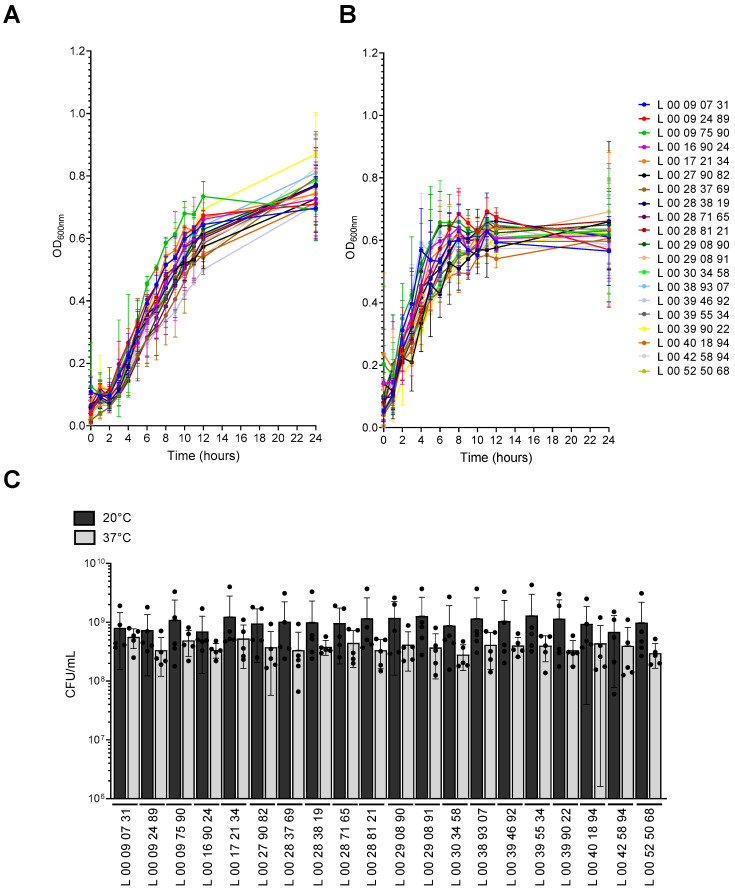
Growth of *V. parahaemolyticus* clinical strains at 20 and 37 °C. Clinical strains of *V. parahaemolyticus* were grown to an optical density at 600 nm (OD_600nm_) of 0.3 in LB2%NaCl at 37 °C. The cultures were diluted in fresh medium and distributed in 96-well culture plates. Bacteria were grown at (**A**) 20 °C or (**B**) 37 °C with agitation. The OD_600nm_ was measured at different times to follow bacterial growth. (**C**) Bacterial counts in colony forming units per ml (CFU/mL) after 24 h of growth. Data are presented as mean ± SD from 5 independent experiments conducted in technical triplicates.

Twenty clinical strains of *V. parahaemolyticus* isolated from sick patients in the province of Quebec, Canada, between 2018 and 2022 were characterized for their virulence and resistance traits at 20 °C and 37 °C. The bacterial growth of the strains at different temperatures was first assessed (Figure 1). Globally, for the same temperature, all the strains grew and exhibited comparable growth curves, as shown by similar OD_600nm_ values through time (Figure 1A,B). Their growth was faster at 37 °C than at 20 °C (Figure 1A,B). At 20 °C, the OD_600nm_ started to increase after 2 h, and while it reached a plateau (OD_600nm_ of 0.6) at 12 h for some strains (4 out 20), it continued to increase until 24 h for the others (Figure 1A). At 37 °C, the growth began as early as 1 h after inoculation (Figure 1B). A plateau was reached for all the strains at an approximative OD_600nm_ of 0.6 after only 8 h (Figure 1B). In contrast to the growth at 20 °C, the OD_600nm_ remained stable after 8 h, and only a slight increase was observed at 24 h (Figure 1B). At 24 h, the OD_600nm_ showed a tendency to be higher at 20 °C than at 37 °C with a mean final OD_600nm_ of 0.756 and 0.621, respectively (Figure 1A,B). Bacterial counts were performed at 24 h of incubation (Figure 1C), and showed a similar tendency of the strains to display a higher number of colony forming units (CFU)/mL at 20 °C than at 37 °C, although this difference was not significant. These results suggest a slower but higher growth at 20 °C than at 37 °C.

### 3.2. Temperature Affects V. parahaemolyticus Sensitivity to Antimicrobials

The resistance of the clinical strains of *V. parahaemolyticus* to antimicrobials was assessed by determination of their MIC for polymyxin B (PmB), carbenicillin, rifampicin, streptomycin, kanamycin, tetracycline, and chloramphenicol (Table 2). These antimicrobials interfere with different essential processes of the bacterial cell, i.e., RNA, cell wall and protein synthesis, and maintenance of membrane integrity. All the strains were sensitive to tetracycline and chloramphenicol, both affecting protein synthesis, regardless of the temperature, with a MIC below 1 µg/mL (Table 2). The strains had a moderate resistance to rifampicin, with MIC values ranging from 3.13 to 12.50 µg/mL (Table 2). For this antibiotic, an increased resistance was noticeable for six strains at 20 °C in comparison to 37 °C but with two strains (L 00 09 24 89 and L 00 39 90 22) being more resistant at 37 °C than at 20 °C. The MIC values of PmB (18/20), carbenicillin (16/20), and streptomycin (13/20) were generally higher at 20 °C than at 37 °C. Regarding kanamycin, the MIC values were sometimes up to four times higher at 37 °C than at 20 °C (Table 2), but five strains were more resistant at 20 °C than at 37 °C.

### 3.3. V. parahaemolyticus Produces More Biofilm at a Lower Temperature

**Figure 2:**
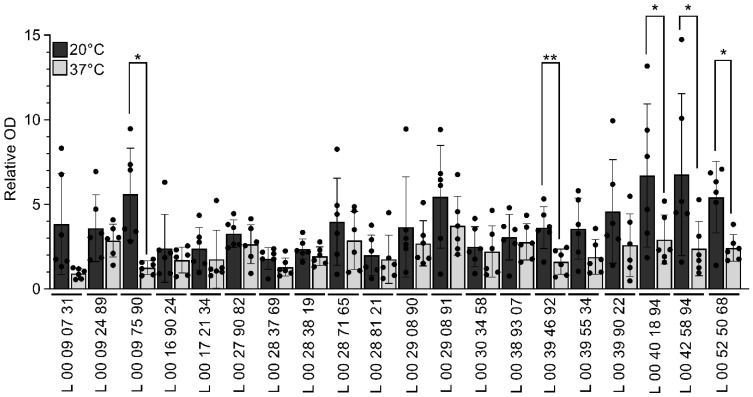
Biofilm production of the clinical strains of *V. parahaemolyticus* at different temperatures. Bacteria were grown in LB2%NaCl at 20 °C (dark bars) and 37 °C (light bars) for 48 h without shaking in 96-well plates. The biofilms were stained with crystal violet and dissolved in acetic acid. The optical density (OD) at 595 nm of the suspension was measured to quantify the biofilm biomass. The relative OD was calculated using the OD at 600 nm of the planktonic phase to normalize biofilm production with bacterial growth. Data are presented as mean ± SD from six independent experiments conducted in technical triplicates. Asterisk represents a significant difference in biofilm biomass quantification for the same strain between growth temperatures, as determined by a one-way ANOVA (*, *p* < 0.05; **, *p* < 0.005).

Because of its importance in environmental persistence, the impact of temperature on *V. parahaemolyticus* clinical strains’ capacity to produce biofilm was assessed (Figure 2). Bacteria were grown in 96-well plates without shaking at 20 °C and 37 °C for 48 h, and the biofilm biomass was quantified using crystal violet. The biofilm production was relativized on OD_600nm_ values to normalize the biofilm quantification on bacterial growth regardless of the effect of temperature on bacterial growth rate.

All strains produced a biofilm at both temperatures (Figure 2). From all the strains, L 00 09 07 31 and L 00 09 75 90 produced the lowest biofilm biomass when grown at 37 °C (Figure 2). L 00 42 58 94 and L 00 40 18 94 produced the highest biomass at 20 °C (Figure 2). Although most strains produced a comparable biofilm biomass at 20 °C (mean = 3.828) and 37 °C (mean = 2.214), some strains produced significantly more biofilm biomass when the bacteria were grown at 20 °C than at 37 °C (Figure 2).

### 3.4. Temperature Has an Impact on V. parahaemolyticus’s Swimming Motility

**Figure 3:**
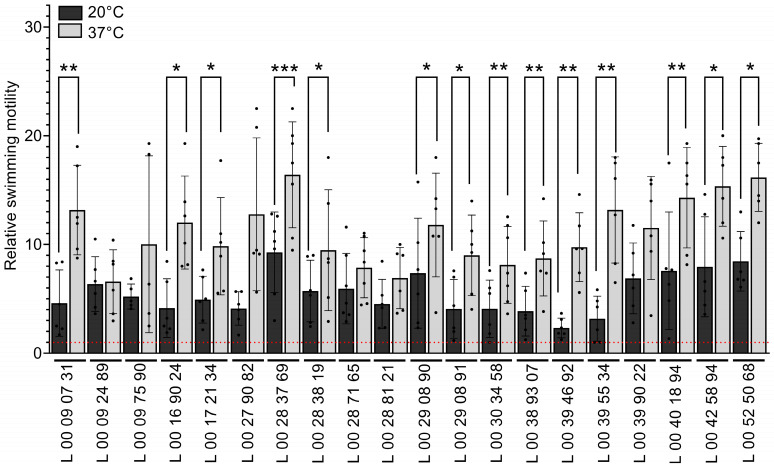
Swimming motility of the clinical strains of *V. parahaemolyticus* at different temperatures. Bacterial cultures were spotted on soft LB2%NaCl—0.3% agar plates and grown at 20 °C (dark grey) and 37 °C (light grey) for 24 h. The diameter of the colony was measured at 24 h on LB2%NaCl with 1.2% agar and 0.3% agar. The colony diameter on 0.3% agar plates was divided by the diameter on 1.2% agar plates. Data are presented as mean ± SD from at least 6 independent experiments. The red dotted line represents a relative motility of 1. Asterisk represents a significant difference in motility for a same strain between growth temperatures, as determined by a one-way ANOVA (*, *p* < 0.05; **, *p* < 0.005; ***, *p* < 0.0005).

Motility, as well as being important for nutrients acquisition through chemotaxis, plays a role in virulence in *V. parahaemolyticus* [24]. The swimming motility of the strains was assessed by measuring the diameter of the colony on motility agar plates at 20 °C and 37 °C after 24 h (Figure 3). All the strains were motile at both temperatures, with a relative motility over 1 (red dotted line) (Figure 3). L 00 28 37 69 showed the highest motility, both at 37 °C and 20 °C, while L 00 39 46 92 showed the lowest at 20 °C (Figure 3). Several strains were significantly more motile at 37 °C than at 20 °C, as determined by a wider colony diameter on 0.3% agar plates, and an overall tendency of increased motility at 37 °C is observed (Figure 3).

### 3.5. Temperature Has No Effect on Membrane Vesicle Formation

**Figure 4:**
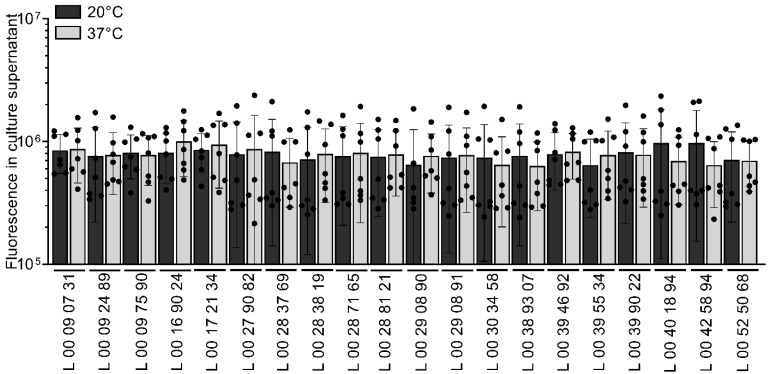
Membrane vesicle quantification in the culture supernatant of clinical strains of *V. parahaemolyticus* grown at different temperatures. Membrane vesicle production in the culture supernatant was assessed using FM1-43. Strains were grown in LB 2% NaCl in 96-well plates at 20 and 37 °C with agitation for 16 h. FM1-43 was added at a final concentration of 10 µg/mL to the culture supernatants, and the fluorescence at 479/579 nm was measured. The relative fluorescence was calculated by dividing the absolute fluorescence values by the optical density at 600 nm of the bacterial cultures. Data are presented as mean ± SD from seven independent experiments conducted in technical duplicates.

Membrane vesicles (MVs) are implicated in various biological functions, including resistance to antimicrobials and phages, membrane modulation, and secretion of virulence factors and misfolded proteins, for example [39,40]. The impact of temperature on MV production was assessed by quantification of the MVs in the culture supernatant after 16 h of growth (Figure 4. MVs were produced by all strains at both temperatures. There was no significant difference in MV production between the strains, as similar fluorescence values were obtained for all the strains (Figure 4). Similar fluorescence values for the same strain between the conditions suggest that MV production was not affected by temperature either in those conditions (Figure 4).

### 3.6. Effect of Temperature on Hemolysin Production

**Figure 5:**
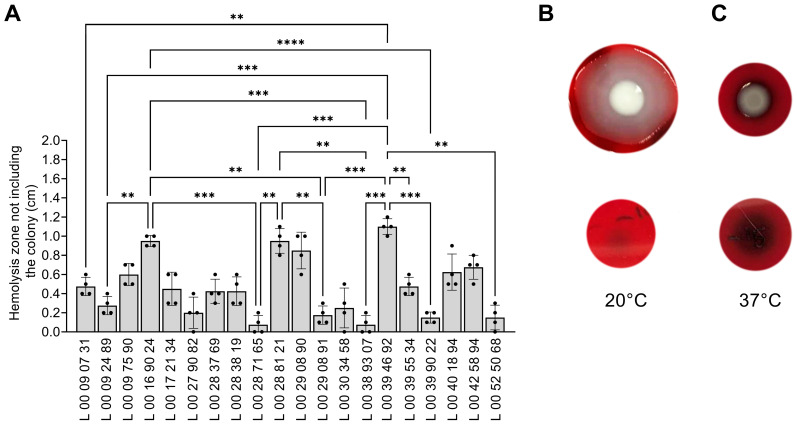
Hemolysin production is affected by temperature. Three microliters of bacterial suspension was spotted on sheep blood agar plates. The plates were incubated at 20 °C or 37 °C for 48 h. (**A**) The width of the hemolysis zone excluding the colony diameter was measured at 37 °C. Data are presented as mean ± SD of four independent experiments. Colony morphology on sheep blood agar after 48 h at (**B**) 20 °C or (**C**) 37 °C (upper panel). The lower-panel pictures show the hemolysis zone under the colonies. Pictures are representative of 3 different experiments. Asterisk represents a significant difference in diameter of the hemolysis zone between the strains, as determined by a one-way ANOVA (**, *p* < 0.005; ***, *p* < 0.0005; ****, *p* < 0.0001).

*V. parahaemolyticus* produces multiple hemolysins, including the pore-forming thermostable direct hemolysin (TDH) and TDH-related hemolysin (TRH) involved in virulence [41]. To assess the impact of temperature on hemolysins production, *V. parahaemolyticus* samples were spotted on TSA sheep blood agar and incubated for 48 h at 20 °C and 37 °C. The diameter of the hemolysis zone was measured (Figure 5A). At 37 °C, all the strains showed a hemolysis zone of variable size and wider than the colony diameter (Figure 5A,C). A variability between the strains was observed, with differences in the width of the hemolysis zone. The mean width of the hemolysis zone, excluding the colony diameter, was 0.466 cm. Interestingly, there was almost no hemolysis at 20 °C for all the strains (Figure 5B, bottom picture), suggesting that the hemolysins are less produced at this temperature. Given the diffused and faint hemolysis, the hemolysis zone could not be measured at 20 °C. Furthermore, at 20 °C, the colony morphology showed large swarming patterns (Figure 5B, top picture). This phenotype was observed at 37 °C, but the colonies were not as outspread as at 20 °C (Figure 5C).

Since *tdh* and *trh* sequences are conserved amongst the strains with more than 80% homology, commonly used sets of primers were used for PCR amplification of those genes to determine their presence in the strains [36]. Fifteen strains contained *tdh*, while nineteen had the *trh* gene (Table 3). The strain L00090731 did not encode for *tdh* or *trh*, although hemolysis was visible on blood agar.

## 4. Discussion

In this study, we characterized twenty strains of *V. parahaemolyticus* isolated from sick patients between 2018 and 2022, including strains from the 2020 outbreak, in the province of Quebec, Canada. Their growth, their capacity to produce biofilm, hemolysins, and MVs, and their motility at 20 °C and 37 °C were assessed to determine their modulation by temperatures found in water and the human body.

First, the bacterial growth at both temperatures was compared. We observed that while the growth was slower, the clinical strains grew at 20 °C in LB2%NaCl and reached a higher final OD_600nm_ than at 37 °C (Figure 1). A previous study showed that *V. parahaemolyticus* did not grow at 21 °C in sea water, while the growth was rapid and began as early as 1 h after the inoculation at 31 °C [42]. These differences could be due to the use of a rich medium instead of sea water, the strains used, or high biofilm production [43]. According to our results, the higher OD_600nm_ after 24 h at 20 °C is likely due to a higher number of bacteria with CFU counts showing a tendency to be higher at this temperature than at 37 °C. After only 1 h of incubation at 37 °C, the OD_600nm_ increased, while at 20 °C, the OD_600nm_ began to rise after 2 h of incubation, suggesting a faster growth at 37 °C. This observation is consistent with previous studies showing a shorter lag time at 37 °C [42,43].

Next, the impact of the temperature on antimicrobial resistance was assessed. The results showed that the clinical strains were resistant to PmB, carbenicillin (β-lactam), the aminoglycosides kanamycin and streptomycin, and, to some extent, rifampicin (rifamycin). Previous studies also reported a high prevalence of resistance to polymyxins, including PmB and colistin, ampicillin (β-lactam), sulfonamide, and streptomycin (aminoglycoside) in *V. parahaemolyticus* clinical strains and strains isolated from seafood [15]. Conversely, all the strains used in this study were sensitive to chloramphenicol and tetracycline. Resistance genes to tetracyclines, β-lactams, aminoglycosides, and rifamycins were found is *V. parahaemolyticus* isolates in a genotypic study and a screening of antimicrobial resistance genes in 10,000 genomes but were not correlated to phenotype [15,44,45].

An impact of temperature on resistance to some antimicrobials was observed. While there was no impact of temperature on resistance to tetracycline and chloramphenicol, the strains had an increased resistance to PmB, carbenicillin, and streptomycin at 20 °C compared to at 37 °C (Table 2). These antibiotics affect different bacterial processes, i.e., the membrane, cell wall, and protein synthesis, respectively. An impact of temperature on antimicrobial resistance was previously observed [46,47,48]. The outer membrane, by being anchored to the peptidoglycan and because of the outer membrane protein and lipopolysaccharide network, is less fluid than the cytoplasmic membrane, conferring resistance to antimicrobials in Gram-negative bacteria [49]. It is known that the temperature affects membrane fluidity, with high temperatures making it more fluid, while low temperatures make it is less fluid [50,51,52]. It was shown in *V. parahaemolyticus* that temperature modifies the relative proportions of saturated and unsaturated fatty acids, affecting membrane fluidity and permeability [53]. A lower temperature might then limit the penetration of antibiotics inside the cell, making the bacteria more resistant to antimicrobials.

Streptomycin is a polycationic antibiotic of the aminoglycoside’s family, which enters the cell after the first interaction with the negatively charged components at the cell surface, such as lipopolysaccharides in Gram-negative bacteria [54]. *Stenotrophomonas maltophilia*, another Gram-negative bacterium, is less susceptible to aminoglycoside antibiotics at 30 °C than at 37 °C because of a reduction in negatively charged phosphate in the lipopolysaccharides at lower temperatures [55]. In other Gram-negative bacteria, low temperature is known to increase the expression of genes from the envelope stress response, leading to resistance to PmB and β-lactam, or to activate alternate sigma factors regulating adaptative responses to environmental stresses [56,57]. Such a response could also occur in *V. parahaemolyticus*, which could explain, at least in part, the resistance to higher PmB and carbenicillin concentrations at 20 °C than at 37 °C.

Overall, our findings show that *V. parahaemolyticus* exhibits increased resistance to several antibiotics at lower temperatures, which could have significant implications for antibiotic use in aquaculture. In these environments, where temperatures are often lower than 37 °C, susceptibility testing performed at standard clinical conditions (37 °C) may underestimate resistance levels seen in the field, leading to inappropriate antibiotic selection or dosing. This mismatch could contribute to the development of antimicrobial resistance in aquaculture settings, where excessive or ineffective use of antibiotics may promote the spread of resistant strains that are more difficult to treat in humans, especially following exposure from seafood or aquaculture sources. Key resistance mechanisms, such as efflux pump activity [58] and β-lactamase production [59], can be thermally regulated, altering their expression depending on the surrounding temperature. However, the overall misuse of antibiotics in these environments—whether due to inadequate dosing or inappropriate drug selection—can drive adaptation in *V. parahaemolyticus*, enhancing its resistance over time. Our current and previous findings [35] emphasize the need for antimicrobial susceptibility testing that considers environmental temperature variations to ensure effective treatment and prevent the emergence of resistant strains.

Lastly, the impact of temperature on the persistence and virulence factors of *V. parahaemolyticus* as it transitions from environmental to human body temperature was assessed. Although an impact of temperature on clinical strains of *V. parahaemolyticus* virulence was observed before, the underlying mechanisms driving this modulation and its effects on virulence and persistence remain unknown [17,42,60].

At 20 °C, representing the marine environment, the strains generally showed more swarming but less swimming motility. *V. parahaemolyticus* possesses a constitutive sheathed polar flagellum, used for swimming motility in liquids, and non-sheathed lateral peritrichous flagella, used for swarming motility on viscous or solid surfaces [61,62,63]. Motility is a key virulence and colonization factor of *V. parahaemolyticus* [62]. Furthermore, both flagellar systems are important for oysters’ colonization and biofilm formation [23,64]. The polar flagellum initiates attachment to surfaces for biofilm formation [65]. The expression of lateral flagella is induced when the rotation of the polar flagellum slows on viscous or solid surfaces [66]. This leads to a differentiation to a swarming phenotype, defined by filamentation of the bacteria and lateral flagella production, used for adhesion to and colonization of surfaces, such as chitin, and microcolony formation with cell-to-cell interaction [61,63,67,68]. The expression of lateral flagella is also modulated by starvation, iron, quorum sensing, and cyclic di-GMP (c-di-GMP) concentrations [66,67]. This correlates with a higher biofilm production at 20 °C than at 37° C, the polar flagellum and biofilm formation being inversely regulated in *V. parahaemolyticus* [62]. For all the strains used in this study, after 48 h, large swarming patterns were observed at 20 °C on sheep blood agar, indicating the production of peritrichous flagella by *V. parahaemolyticus* [69], and although swarming was observed at 37 °C, the diameter was much smaller (Figure 5). These results suggest that *V. parahaemolyticus* might highly produce lateral flagella at 20 °C in specific conditions as this phenomenon was not observed on LB supplemented with 2% NaCl. Conversely to the swarming motility, the clinical isolates of *V. parahaemolyticus* showed generally more swimming motility at 37 °C than at 20 °C (Figure 3), as observed before [17].

Because of the role of the flagella in biofilm formation and the importance of biofilm in *V. parahaemolyticus* environmental persistence, the biofilm biomass was quantified after 48 h, at 20 °C and at 37 °C. A high variability between the strains and an overall higher biofilm production at 20 °C than at 37 °C were observed (Figure 2). *V. parahaemolyticus* biofilm production depends on flagella, capsular polysaccharide production, pili, and adhesion factors such as GbpA [42]. A previous study using 39 environmental and pathogenic strains of *V. parahaemolyticus* isolated from patients or shrimps showed a highly variable strain-dependent biofilm production [70]. A higher global biofilm production and densification were observed at 25 °C than at 37 °C [70]. Other studies showed higher biofilm formation at lower temperatures than at 37 °C [71,72], but the opposite trend can be observed [17,22,42,73] and is likely due to strain variability and experimental conditions. A kinetic analysis of biofilm formation at different temperatures showed that a mature biofilm is obtained faster when incubated at 37 °C (after only 8 h) than at 15 or 25 °C (12 to 48 h) [70]. Biofilm observation after short periods of time would thus lead to a reversed phenotype than after 48 h. Since the biofilm is formed more rapidly at 37 °C than at 20 °C [70], it is possible that after 48 h at 37 °C, the biofilms were in the dispersion phase, reflecting a lower biomass than at 20 °C, for which the mature biofilm is obtained later. Altogether, our results corroborate an impact of temperature on biofilm production in *V. parahaemolyticus*.

Our results also reveal differences in biofilm formation between strains, with some producing two to three times more biomass than others. Several studies have demonstrated the important roles of quorum sensing and c-di-GMP levels in biofilm regulation [74]. *V. parahaemolyticus* produces and responds to several quorum-sensing molecules, including DPO (3,5-dimethylpyrazin-2-ol, produced by the Tdh synthase), AI-1 (N-(3-hydroxybutanoyl)-L-homoserine lactone), AI-2 ((2S,4S)-2-methyl-2,3,3,4-tetrahydroxytetrahydrofuran-borate), and CAI-1 ((Z)-3-aminoundec-2-en-4-one) [75]. Furthermore, quorum sensing plays an important role in c-di-GMP homeostasis in *V. parahaemolyticus* [74,76,77]. Differences in the production and detection of quorum-sensing and c-di-GMP molecules have been reported between *V. parahaemolyticus* strains due to genetic mutations or phase variation [75,78]. It is possible that the variation observed between strains in our study is due to differential expression or functionality of quorum-sensing and c-di-GMP pathways and their downstream effectors, ultimately leading to differences in biofilm formation capacity.

Although they are not essential for virulence [79], TDH and TRH hemolysins are considered the major virulence factor in *V. parahaemolyticus* [41]. Our results showed hemolysis on blood sheep agar plates at 37 °C, but not at 20 °C, by all strains, although a variability in hemolysis between the strains was observed (Figure 5). TDH is a pore-forming toxin that leads to release of water and ions, leading to diarrhea, while TRH is responsible for fluid accumulation in the gut as a result of Cl^−^ release [41]. It was suggested that *V. parahaemolyticus* might express *tdh* and *trh* to acquire nutrients in a hostile environment, such as the host [41]. It was estimated that 10% of clinical isolates do not encode *tdh* and/or *trh*, with some pathogenic isolates lacking both of them [41]. A PCR amplification [36] of *tdh* and *trh* in our 20 clinical strains showed that 95% of the strains had at least one of them, while 75% possessed both genes, which is similar to what was observed. It is, however, possible that because of point mutations, the amplification could not occur on some strains. One strain (L00090731) did not encode for *tdh* or *trh*, although hemolysis was visible on blood agar, with a hemolytic zone near the mean value. However, *V. parahaemolyticus* possesses other hemolytic toxins such as the thermolabile hemolysin *tlh* and δ-VPH, which could explain this phenotype [80,81,82]. The strains L00394692 and L00169024 had the widest hemolytic zones, even without encoding for *tdh*, suggesting a differential regulation in those strains. Previous studies reported a temperature effect on hemolysins, as the expression of *tdh* and the global hemolytic activity of *V. parahaemolyticus* were higher at 37 °C than at lower temperatures [17,42,83], which correlates with our results.

Taken together, our results clearly demonstrate an impact of temperature on persistence and virulence factors produced by *V. parahaemolyticus*, which can provide colonization (20 °C) or virulence (37 °C) advantages (Table 4). Some phenotypes at 20 °C could help *V. parahaemolyticus* to persist in oysters and in the aquatic environment, such as swarming, which is associated with high adherence and biofilm formation [61], and biofilm formation that protects the bacteria from hostile environment [65]. The strains were also more resistant to PmB, a cationic antimicrobial peptide, at 20 °C. Although PmB is not produced by oysters, cationic antimicrobial peptides play a crucial role in their immune defense system [84]. Conversely, at 37 °C, the strains grew faster and produced virulence-associated factors like TDH and TRH, and a high swimming motility. TDH and TRH are toxins with cytotoxic and hemolysin activities and are associated with pathogenic strains [41].

Bacterial pathogens that alternate between environmental reservoirs and mammalian hosts often rely on environmental signals to regulate the expression of virulence-related genes [85]. In *V. parahaemolyticus*, clinical and environmental strains exhibit notable differences in virulence gene expression, with clinical strains showing increased activation of virulence factors—including hemolysins, biofilm formation, and motility—when shifting from 28 °C to 37 °C [17]. Several regulators mediate the temperature-dependent expression of virulence effectors in *V. parahaemolyticus*. Notably, the histone-like nucleoid-structuring protein (H-NS) contributes to the activation of T3SS2—an apparatus involved in TDH secretion—at 37 °C but not at temperatures below 30 °C, including 20 °C [83,86]. This regulation pattern aligns with our hemolytic activity results, although additional hemolysins are likely involved.

Another study showed that a temperature shift from 16 °C to 30 °C induces the expression of flagellar assembly genes, as well as genes encoding the type VI secretion system (T6SS) and adhesion factors [87]. In their analysis, both *opaR* and *toxR*—two known regulators—were downregulated at 30 °C, consistent with T6SS activation. Although ToxR is a positive regulator of motility and its deletion reduces motility [88], an increased motility at 37 °C was observed here. OpaR is a known repressor of polar flagellum expression [89]. Thus, the downregulation of *opaR* at 30 °C may partly explain the upregulation of flagellar-related genes, which aligns with the increased motility we observed.

OpaR, the master regulator of quorum sensing in *V. parahaemolyticus*, plays a crucial role in the regulation of biofilm formation. This process is highly controlled and influenced by the secondary messenger c-di-GMP in a strain-dependent manner. OpaR can function either as a repressor or an activator of biofilm formation. As a repressor, it reduces intracellular c-di-GMP levels, thereby inhibiting biofilm development [76]. Conversely, it can also promote biofilm formation by upregulating the expression of *cpsQ*, which encodes a c-di-GMP-binding protein that activates this process [90,91]. A previous study showed that biofilm formation in *V. parahaemolyticus* is influenced by temperature. Thicker biofilms were observed at lower temperatures (15 °C and 25 °C) compared to 37 °C, with the densest biofilm forming at 25 °C [70], consistent with our observations.

Given the expected rise in *Vibrio* species, including pathogens, in marine environments due to warming temperatures, especially in coastal areas where shellfish are harvested, it may become necessary to combine multiple post-harvest methods for more effective control of *V. parahaemolyticus*. A systematic review by Spaur and colleagues [92] evaluated several post-harvest interventions for *V. parahaemolyticus* in raw oysters and assessed their effectiveness in reducing bacterial contamination. They found that high hydrostatic pressure was the most consistently effective method, achieving significant bacterial reductions without compromising oyster quality. Other approaches, such as freezing, depuration, and high-salinity relaying, showed variable effectiveness, often influenced by environmental conditions or negative impacts on oyster sensory attributes. In all cases, strict maintenance of the cold chain after harvesting is essential to prevent bacterial proliferation. Studies show that 18% of oyster shipments exceed the temperature guidelines set by the U.S. government, while proper cold chain management reduces *V. parahaemolyticus* levels in 75% of shipments [93]. Notably, temperatures between 15 and 20 °C are commonly observed between harvest and shipping. In our study, we observed that biofilm formation at 20 °C enhances bacterial survival and increases resistance to environmental stresses. Therefore, maintaining post-harvest temperatures well below this threshold is crucial not only to limit bacterial growth but also to prevent the formation of biofilms, which can reduce the effectiveness of downstream interventions. These findings emphasize the importance of integrating temperature control with sanitation measures and, where appropriate, post-harvest treatments to safeguard seafood safety especially in the context of a warming climate. With the increased prevalence of *V. parahaemolyticus* infections due to climate change, a better comprehension of this pathogen and the regulation of its virulence and persistence in changing environmental conditions will also be necessary to prevent and limit infections and ensure food safety.

## Figures and Tables

**Table 1:** Bacterial strains used in this study.

Strain	Sampling Date
*V. parahaemolyticus*	
L 00 09 07 31	August 2018
L 00 09 24 89	August 2018
L 00 09 75 90	September 2018
L 00 16 90 24	August 2019
L 00 17 21 34	September 2019
L 00 27 90 82	July 2020
L 00 28 37 69	August 2020
L 00 28 38 19	August 2020
L 00 28 71 65	September 2020
L 00 28 81 21	September 2020
L 00 29 08 90	September 2020
L 00 29 08 91	September 2020
L 00 30 34 58	October 2020
L 00 38 93 07	September 2021
L 00 39 46 92	September 2021
L 00 39 55 34	September 2021
L 00 39 90 22	October 2021
L 00 40 18 94	October 2021
L 00 42 58 94	December 2021
L 00 52 50 68	August 2022

**Table 2:** Minimal inhibitory concentration (MIC) of antimicrobial peptides and antibiotics at 20 °C and 37 °C for all the clinical strains of *V. parahaemolyticus*.

		MIC (µg/mL)													
	ATB	PmB		Carb		Rifampicin		Strep		Kana		Tetra		Chloramp	
	Target	Membrane		Cell Wall		RNA Synthesis		Protein Synt.		Protein Synt.		Protein Synt.		Protein Synt.	
	T°	20 °C	37 °C	20 °C	37 °C	20 °C	37 °C	20 °C	37 °C	20 °C	37 °C	20 °C	37 °C	20 °C	37 °C
Strain	Sampling Date														
L 00 09 07 31	August 2018	50	25	100	50	6.25	6.25	25	25	25	12.5	1.56	0.78	0.78	0.78
L 00 09 24 89	August 2018	100	50	100	50	6.25	12.5	50	25	25	50	1.56	0.78	0.78	0.78
L 00 09 75 90	September 2018	50	50	100	50	6.25	6.25	50	25	25	100	0.78	0.78	0.78	0.78
L 00 16 90 24	August 2019	50	25	100	50	6.25	6.25	50	25	25	12.5	0.78	0.78	0.78	0.78
L 00 17 21 34	September 2019	50	25	100	50	6.25	6.25	50	50	25	25	0.78	0.78	0.78	0.78
L 00 27 90 82	July 2020	100	50	50	50	6.25	6.25	25	50	25	50	0.78	0.78	0.78	0.78
L 00 28 37 69	August 2020	100	50	50	50	6.25	6.25	50	25	25	25	0.78	0.78	0.78	0.78
L 00 28 38 19	August 2020	100	50	50	25	12.5	3.13	50	25	25	25	0.78	0.78	0.78	0.78
L 00 28 71 65	September 2020	100	50	50	50	6.25	3.13	50	25	25	25	0.78	0.78	0.78	0.78
L 00 28 81 21	September 2020	100	50	50	50	6.25	3.13	50	50	25	25	0.78	0.78	0.78	0.78
L 00 29 08 90	September 2020	100	50	100	50	6.25	3.13	50	25	25	25	0.78	0.78	0.78	0.78
L 00 29 08 91	September 2020	100	50	100	25	6.25	3.13	50	25	25	25	0.78	0.78	0.78	0.78
L 00 30 34 58	October 2020	100	50	50	25	6.25	6.25	50	50	25	50	0.78	0.78	0.78	0.78
L 00 38 93 07	September 2021	100	100	50	25	6.25	6.25	50	25	50	12.5	0.78	0.78	0.78	0.78
L 00 39 46 92	September 2021	50	50	100	50	6.25	3.13	25	25	12.5	6.26	0.78	0.78	0.78	0.78
L 00 39 55 34	September 2021	100	50	100	50	6.25	6.25	50	50	25	25	0.78	0.78	0.78	0.78
L 00 39 90 22	October 2021	100	50	100	50	6.25	12.5	50	25	25	100	0.78	0.78	0.78	0.78
L 00 40 18 94	October 2021	100	50	100	50	6.25	6.25	50	25	25	50	0.78	0.78	0.78	0.78
L 00 42 58 94	December 2021	100	50	100	50	6.25	6.25	50	25	25	50	0.78	0.78	0.78	0.78
L 00 52 50 68	August 2022	100	50	100	25	6.25	6.25	50	25	50	12.5	0.78	0.78	0.78	0.78

Legend: ATB, antibiotic; Target, target of the antibiotic; Carb, carbenicillin; Chloram, chloramphenicol; Kana, kanamycin; MIC, minimal inhibitory concentration; PmB, polymyxin B; Strep, streptomycin; synt., synthesis; T°, growth temperature; Tetra, tetracycline. A bright red shade highlights a higher MIC value to a given antimicrobial than lighter shades.

**Table 3:** Presence of thermostable direct hemolysin (TDH) and TDH-related hemolysin (TRH) in the strains as determined by PCR amplification.

Strain	TDH	TRH
L 00 09 07 31	−	−
L 00 09 24 89	+	+
L 00 09 75 90	−	+
L 00 16 90 24	−	+
L 00 17 21 34	−	+
L 00 27 90 82	+	+
L 00 28 37 69	+	+
L 00 28 38 19	+	+
L 00 28 71 65	+	+
L 00 28 81 21	+	+
L 00 29 08 90	+	+
L 00 29 08 91	+	+
L 00 30 34 58	+	+
L 00 38 93 07	+	+
L 00 39 46 92	−	+
L 00 39 55 34	+	+
L 00 39 90 22	+	+
L 00 40 18 94	+	+
L 00 42 58 94	+	+
L 00 52 50 68	+	+

+, amplification; −, no amplification.

**Table 4:** Summary of the temperature effects on clinical strains of *V. parahaemolyticus*.

	20 °C	37 °C
Growth		
Speed	Slow	Fast
Maximum OD_600nm_	High	Low
CFU/mL	High	Low
Biofilm biomass	High	Low
Motility		
Swimming	Low	High
Swarming	High	Low
Resistance to antimicrobials	More resistant	Less resistant
Hemolysis	None	Yes

## Data Availability

The original contributions presented in this study are included in the article. Further inquiries can be directed to the corresponding author.
